# Common variation in the *SERPING1* gene is not associated with age-related macular degeneration in two independent groups of subjects

**Published:** 2009-01-23

**Authors:** Kyu Hyung Park, Euijung Ryu, Nirubol Tosakulwong, Yanhong Wu, Albert O. Edwards

**Affiliations:** 1 Department of Ophthalmology, Mayo Clinic, Rochester, MN; 2 Department of Ophthalmology, Seoul National University, Bundang Hospital, Gyeonggi, Korea; 3 Division of Biomedical Statistics and Informatics, Mayo Clinic, Rochester, MN; 4 Department of Laboratory Medicine and Pathology, Mayo Clinic, Rochester, MN

## Abstract

**Purpose:**

Common genetic variation in the complement component 1 inhibitor gene (*SERPING1*) was recently reported to increase the risk of developing age-related macular degeneration (AMD). This study was performed to replicate the association between *SERPING1* and AMD.

**Methods:**

Seven single nucleotide polymorphisms (SNPs) tagging common haplotypes across *SERPING1* were genotyped on 786 (The Mayo Clinic) subjects and the association with AMD studied using single SNP and haplotype association analyses. The SNP in intron 6 (rs2511989) previously reported to increase the risk of AMD was studied in an additional 1,541 subjects from the Age-Related Eye Disease Study (AREDS). Association with specific subtypes of AMD and interaction with four other loci: complement factor H (*CFH*), age-related maculopathy susceptibility 2 (*ARMS2*/*LOC387715*), High Temperature Requirement Factor A1 (*HTRA1*), complement factor B/complement component 2 (*CFB/C2*), and complement component 3 (*C3*) involved in AMD was explored.

**Results:**

The seven tag-SNPs were not associated with AMD in the Mayo subjects (p=0.13–0.70) and rs2511989 was also not associated with AMD in the Mayo or AREDS subjects (p=0.44–0.45). Evaluation of haplotypes across *SERPING1* did not reveal association with AMD (p=0.14–0.97). SNPs were not associated with AMD subtypes (early, geographic atrophy, or exudation). No interaction with other AMD risk variants was observed.

**Conclusions:**

We were unable to replicate the reported association between *SERPING1* and AMD in two independent groups of subjects.

## Introduction

Age related macular degeneration (AMD) is a leading cause of irreversible loss of vision in older individuals [1]. Both genetic and cell biologic analyses support a role for dysregulation of innate immunity in the pathogenesis of AMD. Extensive deposition of innate immunity proteins at the level of Bruch’s membrane [2,3] in AMD patients has been consistently observed. Genetic variants in complement pathway genes including complement factor H (*CFH)* [4-6], complement factor B (*CFB)/*complement component 2 *(C2)* [7], and complement component 3 (*C3)* [8-11] are established risks for developing AMD. The association between AMD and genetic variation in these loci has been extensively replicated as recently reviewed [12]. Many other common genetic variants have been proposed for AMD. However, review of a partial list shows that they do not yet have sufficient support due to the absence of replication studies [13-20] or failure to consistently replicate the genetic association in independent groups of subjects [21-29]. Thus, at this time there is strong evidence for the involvement of genetic variation in genes of the alternative pathway of complement and one additional locus (*LOC387715/*High Temperature Requirement Factor A1 [*HTRA1*]) on chromosome 10q26 [30,31].

Recently, there has been interest in exploring the genes involved in classical complement activation initiated by antibody-antigen interactions. Evidence for a possible role of the classical complement pathway comes from the observation of anti-retinal antibodies in patients with AMD [32-35], animal models with AMD-like pathology [36], and expression of these proteins in the aging retina and retinal pigment epithelium (RPE) [37].

The complement component 1 (C1) inhibitor is a key regulator of the classical pathway and has been reported to down-regulate the alternative pathway in vitro by binding to C3b and inhibiting binding of complement factor B to C3b [38]. The C1 inhibitor has sequence homology with serine protease inhibitors (SERPIN) and inhibits activation of the classical and lectin compliment pathways by inhibiting the protease activity of complement component 1, r subcomponent (C1r), complement component 1, s subcomponent (C1s), and mannan-binding lectin serine peptidase 2 (MASP-2) [39,40]. The C1 inhibitor also regulates vascular permeability by inhibiting proteases that generate bradykinin [40], as manifested when deficiency of C1 inhibitor results in hereditary angioedema [41-43]. Thus, C1 inhibitor is an excellent candidate gene for involvement in AMD and retinal aging.

Since the discovery of the association between AMD and variants in *CFH*, the systematic study of other genes regulating or involved in the alternative pathway of complement activation has been productive (e.g., the study of *CFB/C2*, *C3* [7-11]). The systematic study of other genes involved in innate immunity is ongoing in several laboratories. A recent study reported a protective effect on AMD for the minor allele of a SNP (rs2511989) within intron 6 of the *SERPING1* gene encoding the C1 inhibitor [13]. The purpose of this study was to replicate the association between *SERPING1* and AMD in two independent groups of subjects. We performed a systematic analysis of all common haplotypes (ancestral segments of DNA inherited as a block in a population) across the *SERPING1* locus and identified no evidence for association with AMD.

## Methods

### Subjects

The study was approved by the institutional review board of the Mayo Clinic (Rochester, MN) and written informed consent was obtained from all subjects. The Mayo subjects were composed of 786 Caucasian individuals (476 AMD cases, 310 controls without AMD). Subjects and cases were obtained from the same eye clinics of participating physicians. Diagnosis was determined by review of fundus photographs as described previously [4,44,45]. Briefly, all subjects diagnosed with AMD had large drusen (≥125 microns) with sufficient drusen area to fill a 700 micron circle or more advanced findings. Controls had 5 or fewer hard drusen (<63 microns) without pigment changes or more advanced findings. Geographic atrophy and exudation were defined using the Wisconsin age-related maculopathy grading system [46]. The Mayo subjects have been graded multiple independent times by Dr. Edwards and were recently re-graded by Dr. Park. Replication studies were performed on 1,541 Caucasian subjects (1,241 with AMD and 300 controls without AMD) from the Age-Related Eye Disease Study (AREDS) that were graded as reported previously [47]. The final AREDS phenotype grade was used. All control grades (controls and control questionable 1–4) were treated as controls. Early AMD consisted of all grades of large drusen (large drusen and large drusen questionable 1–3). Review of genotype frequencies for the control questionable and large drusen questionable AREDS subjects at known AMD risk variants showed that the questionable groups closely matched their assigned group (e.g., for the *C3* SNP rs2230199, genotype frequencies were within 1% when the questionable control or large drusen categories were excluded). Further, the maximal difference in genotype frequencies for the *SERPING1* SNP rs2511989 was 0.03.

Advanced AMD AREDS subjects consisted of all advanced AMD grades (questionable advanced, neovascular, geographic atrophy, and both neovascular and geographic atrophy). Mayo or AREDS subjects with both neovascular and geographic atrophy were included in the analysis for each subtype, except when the analytical model required a unique grade. When a unique grade for each subject was required, the subjects graded both were added to the grade with a smaller number of subjects (geographic atrophy) to increase power. Individual SNP analyses were done with and without both, and no differential effect was observed. Eighty-seven subjects with the questionable advanced AMD grade only were used for any AMD versus control analyses. Note that the number of subjects shown in the tables may be less than the total number of subjects available for study, due to failed genotyping. No detectable substructure has been observed within the Mayo subjects that might inflate case-control statistics, and the small amount of sub-structure within the AREDS subjects had no effect on the individual SNP case-control statistics. Demographic and phenotypic information for the Mayo and AREDS subjects is provided in Table 1.

**Table 1:** Demographic and phenotypic features of the Mayo and AREDS subjects

**Category**	**Mayo subjects**			**AREDS subjects**		
**Number**	**Age (Mean±SD)**	**Male:Female ratio**	**Number**	**Age (Mean±SD)**	**Male:Female ratio**
Control subjects	310	69.5±8.2	0.83	300	77.6±4.3	0.79
AMD subjects*	476	76.9±9.6	0.55	1241	79.9±5.1	0.68
Early AMD	218	73.7±10.3	0.46	583	79.0±4.9	0.65
Exudation	161	79.2±8.7	0.59	324	80.6±5.0	0.76
Geographic atrophy**	97	80.2±6.6	0.70	247	80.8±5.3	0.65

*Eighty-seven AREDS subjects with the “questionable advanced age-related macular degeneration (AMD)” grade were used as described in Methods, but are not included in this table. **29 Mayo subjects and 85 AREDS subjects who had both geographic atrophy and exudative AMD (category “Both”) were included in the geographic atrophy group as described in the Methods. Abbreviations: SD is standard deviation.

### Selection of tag and functional SNPs

SNPs genotyped in the international haplotype map (HapMap) [48] were used to select tag SNPs. SNPs across *SERPING1* including 5 kb up and downstream were evaluated for linkage disequilibrium (LD) using ldSelect [49] and a custom algorithm for tag-SNP selection was developed so that a single tag SNP would be selected for each LD bin. Only those SNPs deemed candidate tag-SNPs by ldSelect with a minor allele frequency (MAF) of greater than or equal to 0.05 were considered for further selection. Tag-SNPs were ultimately selected based upon a functional ranking system wherein non-synonymous coding SNPs were preferentially selected among the tag-SNP candidates in each LD bin, followed by synonymous coding SNPs, SNPs from 5′ untranslated regions (UTRs), SNPs from 3′ UTRs, and finally SNPs from intronic regions. Seven SNPs were required to tag the *SERPING1* haplotypes and were selected for genotyping.

### Genotyping

The seven tag-SNPs were genotyped on genomic DNA extracted from peripheral blood leukocytes using TaqMan assays. Clustering of genotypes was inspected for separation between genotypes and accuracy of genotype calls. Quality control for genotype call was assessed by concordance for the control CEPH trio DNA replicates and two sample replicates within each 96 well plate. Randomly selected cases (25 subjects) and controls (25 subjects) were subjected to bi-directional DNA sequencing to determine the accuracy of genotyping with rs2511989 in a masked fashion.

### Statistics

Upon receipt of genotype intensities and processing as described above, all SNPs were noted to be in Hardy–Weinberg equilibrium. Single SNP analyses on genotype distributions were performed in SAS version 8 (SAS Institute, Cary, NC) using logistic regression assuming a log-additive genetic model where SNPs were coded as 0, 1, or 2 for the number of minor alleles and the corresponding p-values were calculated based on the score test statistics. Fisher’s exact tests were also performed on genotype distributions. Haplotype analyses were completed using the score test that consisted of the seven SNPs across *SERPING1.* We also performed haplotype analysis with a 3-SNP-sliding-window approach, as implemented in haplo.stats [50] using R statistical software. Interaction effect between *SERPING1* SNPs and smoking status (defined as ever, never) on AMD status was evaluated under a log-additive model with logistic regression using two main effect terms (genotype for each SNP and smoking status) and their interaction term, and assessed by a likelihood ratio test. We also evaluated interaction between *SERPING1* SNPs and other major genetics risks for AMD including complement factor H (*CFH*, Y402H), *LOC387715*/*HTRA1* (tagging SNP A69S), *CFB/C2* (*CFB*, L9H; *C2*, rs547154), and *C3* (R102G and P314L). To investigate the effect of the seven SNPs of *SERPING1* including rs2511989 on AMD subtypes, likelihood ratio tests were performed considering controls as baseline. Haplotype analyses for AMD subtypes were also performed using a likelihood ratio test. Age is confounded with diagnosis (i.e., the cases are older than the controls), thus correction for age might reduce any detectable genetic effect and was not employed. Nominal p-values are reported.

## Results

### Association between individual SNPs and AMD in the Mayo subjects

None of the seven SNPs tagging *SERPING1* haplotypes (including rs2511989) were associated with AMD subjects compared to controls without AMD (Table 2). Because of the previously reported association between rs2511989 and AMD, we sought to determine the accuracy of genotyping using DNA sequencing. The genotype for 50 subjects determined by DNA sequencing was in complete agreement with the TaqMan assay genotype calls. Thus, the absence of association could not be explained by inaccurate genotyping.

**Table 2:** Distribution of genotypes for the seven tag-SNPs across *SERPING1* and their association with AMD in the Mayo subjects

**SNP**	**Function**	**Location**	**Minor allele frequency**	**Hardy–Weinberg equilibrium (p-value)**	**Genotype**	**Number AMD Cases (%)**	**Number controls (%)**	**Fisher p-value**	**Log-additive p-value**
rs2509897	2.4 K upstream	57119193	0.36	0.87	GG	197 (42)	122 (39)	0.66	0.52
					GC	204 (44)	146 (47)		
					CC	64 (14)	42 (14)		
rs2511990	2.3 K upstream	57119284	0.5	0.19	TT	126 (27)	80 (26)	0.72	0.51
					TC	223 (48)	141 (46)		
					CC	116 (25)	84 (28)		
rs3758919	2.2 K upstream	57119379	0.11	0.92	CC	382 (81)	236 (77)	0.24	0.13
					CT	80 (17)	67 (22)		
					TT	7 (1)	5 (2)		
rs1005511	Intron 2	57123232	0.33	0.26	GG	208 (46)	124 (43)	0.43	0.7
					GA	194 (43)	137 (47)		
					AA	52 (11)	28 (10)		
rs2508443	Intron 6	57134568	0.4	0.37	GG	180 (38)	104 (34)	0.27	0.47
					GT	211 (45)	157 (51)		
					TT	78 (17)	48 (16)		
rs2511989	Intron 6	57134901	0.4	0.27	GG	179 (38)	103 (33)	0.27	0.44
					GA	211 (45)	157 (51)		
					AA	80 (17)	50 (16)		
rs4926	V480M (Exon 8)	57138565	0.27	0.52	GG	252 (54)	167 (54)	0.14	0.36
					GA	172 (37)	124 (40)		
					AA	47 (10)	19 (6)		

The seven single-nucleotide polymorphisms (SNPs) were tested for association with age-related macular degeneration (AMD) in the Mayo subjects and none was observed.

### Linkage disequilibrium

**Figure 1:**
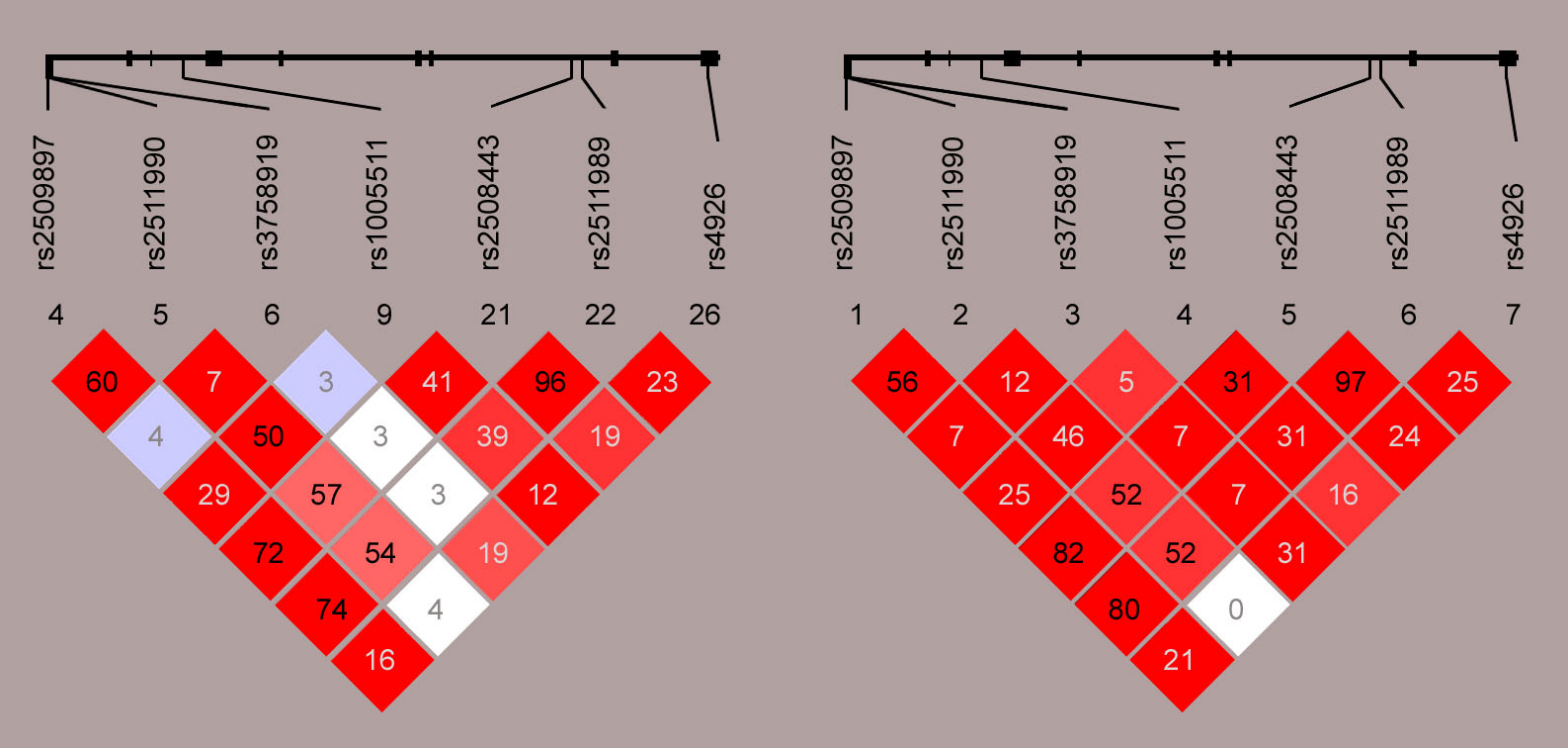
Linkage disequilibrium (LD) across the *SERPING1* locus in HapMap (left) and Mayo subjects (right). A similar LD pattern was observed for the two independent groups of Caucasian subjects. Numbers in the squares represent r^2^ estimates, while colors represent D’ estimates (from none or white, to complete or red).

To verify that our genotyping results were similar to other Caucasian populations genotyped across *SERPING1*, we compared the pattern of linkage disequilibrium (LD) in the Mayo subjects to the international HapMap Caucasian subjects using Haploview [48,51]. Moderate to high LD was observed across the *SERPING1* locus and a similar pattern was present in the Mayo subjects and HapMap Caucasian subjects (Figure 1). The similar pattern of LD further supports the accuracy of genotyping of all seven SNPs in the Mayo subjects.

### Haplotype studies

Comparison of haplotypes across the *SERPING1* locus in the Mayo subjects and the international HapMap Caucasian subjects showed a similar distribution of haplotypes (Table 3). Further, no haplotype was associated with AMD in the Mayo subjects (p=0.14–0.97, Table 3). A 3-SNP sliding window analysis did not reveal association between the *SERPING1* haplotypes and AMD (p=0.13–0.67).

**Table 3:** *SERPING1* haplotypes observed in the Mayo subjects and Caucasian subjects from the International HapMap project

Haplotype	Frequency			Association with AMD in Mayo subjects	
	HapMap	Mayo cases	Mayo controls	Odds ratio (95% CI)	Simulated p-value
GTCGTAG	0.395	0.347	0.368	Reference	0.436
CCCAGGG	0.325	0.320	0.317	1.064 (0.828–1.368)	0.966
CTCGGGA	0.071	0.127	0.102	1.330 (0.934–1.894)	0.137
CCCGGGA	0.062	0.098	0.120	0.902 (0.642–1.267)	0.189

Haplotypes with a frequency of 5% or higher are shown. The single-nucleotide polymorphism (SNP) order (left to right) is the same as in Table 1. Haplotypes across the *SERPING1* locus were tested for association with age-related macular degeneration (AMD) in the Mayo subjects and none was observed.

### Replication study

Given the demonstrated accuracy of genotyping, we sought to determine if the absence of association could be due to a type II error arising in the Mayo subject group. The most highly associated variant in the previous report (rs2511989) was genotyped on 1,541 AREDS subjects (Table 4) and no association with AMD was observed (p=0.45).

**Table 4:** Comparison of the association observed between AMD and the *SERPING1* variant rs2511989 in four subject groups

**Subject group (genotyping platform)**	**Mayo subjects (TaqMan)**		**AREDS subjects (TaqMan)**		**Ennis et al. (UK) subjects (Illumina)**		**Ennis et al. (US) subjects (TaqMan)**	
**Subjects**	**Case**	**Control**	**Case**	**Control**	**Case**	**Control**	**Case**	**Control**
No. of participants	470	310	1221	295	479	479	248	252
								
Allele count (%)								
G	569 (61)	363 (59)	1435 (59)	357 (61)	597 (63)	500 (52)	322 (65)	282 (56)
A	371 (39)	257 (41)	1007 (41)	233 (39)	355 (37)	454 (48)	174 (35)	222 (44)
								
Genotype count (%)								
GG	179 (38)	103 (33)	436 (36)	115 (39)	191 (40)	132 (28)	100 (40)	79 (31)
GA	211 (45)	157 (51)	563 (46)	127 (43)	215 (45)	236 (50)	122 (49)	124 (49)
AA	80 (17)	50 (16)	222 (18)	53 (18)	70 (15)	109 (23)	26 (11)	49 (19)
								
P-values								
Allele	0.46	–	0.41	–	5.4E-06	–	0.0037	–
Genotype	0.44	–	0.45	–	4.0E-05	–	0.0080	–

Allele frequencies and genotype counts are shown for all four subject groups genotyped on rs2511989 to date. The differences between cases and controls in the Mayo and AREDS subjects did not reach statistical significance.

### AMD subtype analysis

To determine if the absence of association between AMD and rs2511989 could be due to differences in the distribution of AMD subtypes in the different subject groups, we stratified the AMD subjects into early AMD, primary geographic atrophy, and exudation. Subgroup analysis using the seven tag-SNPs genotyped on the Mayo subjects revealed no evidence for association with any AMD subtype (early AMD, p=0.43–0.88; geographic atrophy, p=0.13–0.96; exudation, p=0.05–0.66). Similarly, no association between rs2511989 genotypes and AMD subtypes were observed in the AREDS subjects (p=0.17–0.97). No haplotype was consistently associated with any AMD subtype (p=0.09–0.98).

### Interaction with other genetic risks for AMD

We further sought to determine if the absence of an effect of rs2511989 on AMD risk might be explained by differences between modifiable risk factors and other genetic risks for AMD in the Mayo subjects. We observed no interaction between smoking categorized as ever or never and *SERPING1* SNPs using logistic regression (p=0.25–0.52). We also found no interaction (p=0.68–0.98) between rs2511989 and other major genetics risks for AMD including complement factor H (*CFH*, Y402H), *ARMS2/HTRA1* (tagging SNP A69S), complement components *CFB/C2* (*CFB*, L9H; *C2*, rs547154), and *C3* (R102G and P314L).

## Discussion

We were unable to replicate the previously reported association between *SERPING1* and AMD using two independent groups of subjects [13]. Our study comprehensively assessed common variation in *SERPING1* and specifically the SNP (rs2511989) most highly associated with AMD in the previous study [13].

The previous study observed several SNPs (rs2244169, rs2511990, rs2509897, rs2511989, rs2511988, and rs1005510) across *SERPING1* that were associated with AMD [13]. We genotyped three of these SNP (rs2509897, rs2511990, and rs2511989) and observed no association with AMD. Although some investigators have felt that observing association from SNPs near the initial disease associated SNP provides additional support for the association, we have observed otherwise [11]. This situation arises secondary to linkage disequilibrium and supports accurate genotyping, but does not protect against a type I error [11].

We recognize that a particular group of subjects may miss a valid association (type II error) or show false association (type I error). Several causes for such observations exist including invalid genotyping, population stratification, non-random sampling of the underlying population, and differences in non-randomized features of the cases and controls such as disease subtypes. For these reasons, we validated the genotyping with a second independent assay, studied two independent populations, and extensively studied the disease subgroups and similarities between our Caucasian populations and those genotyped elsewhere (HapMap). All of these analyses supported the validity of our study and failed to provide evidence for an effect of *SERPING1* SNPs on AMD. Genotyping of additional groups of subjects will be required to determine if *SERPING1* SNPs are associated with AMD in selected populations. Nonetheless, a major effect on AMD is unlikely.

Genotyping errors can reduce power and are thus of particular concern in a study that fails to replicate a previous observation. The previous study used an Illumina BeadChip platform in one group of subjects and an assay on demand TaqMan method in a second independent group of subjects [13] for genotyping rs2511989 (Table 4). Eight additional SNPs were genotyped using a third (KBioscience, Hoddesdon, UK) platform. Thus, genotyping errors seem unlikely to have caused a type I error in the previous report. We employed the same genotyping assay (TaqMan, Foster City, CA) used on one of their subject groups on both of our groups of subjects and performed DNA sequencing to confirm genotyping results. Genotyping problems seem unlikely to explain the differences between the studies.

*SERPING1* is expressed in the neural retina, RPE, and choroid of humans [13] and is likely to play a role in regulating the complement system in the eye. Mutations in *SERPING1* cause hereditary angioedema, but there is no known phenotypic overlap with AMD such as had been established with atypical hemolytic uremic syndrome or glomerulonephritis arising in patients with mutations in *CFH* [52]. In summary, we were unable to replicate the association between genetic variation in *SERPING1* and AMD in two large and well characterized Caucasian subject groups and no compelling biologic evidence is available to support a role for the C1 inhibitor in AMD at this time.
